# Isotopic signatures of methane emissions from tropical fires, agriculture and wetlands: the MOYA and ZWAMPS flights

**DOI:** 10.1098/rsta.2021.0112

**Published:** 2022-01-24

**Authors:** MOYA/ZWAMPS Team, Euan G. Nisbet, Grant Allen, Rebecca E. Fisher, James L. France, James D. Lee, David Lowry, Marcos F. Andrade, Thomas J. Bannan, Patrick Barker, Prudence Bateson, Stéphane J.-B. Bauguitte, Keith N. Bower, Tim J. Broderick, Francis Chibesakunda, Michelle Cain, Alice E. Cozens, Michael C. Daly, Anita L. Ganesan, Anna E. Jones, Musa Lambakasa, Mark F. Lunt, Archit Mehra, Isabel Moreno, Dominika Pasternak, Paul I. Palmer, Carl J. Percival, Joseph R. Pitt, Amber J. Riddle, Matthew Rigby, Jacob T. Shaw, Angharad C. Stell, Adam R. Vaughan, Nicola J. Warwick, Shona E. Wilde

**Affiliations:** ^1^ Department of Earth Sciences, Royal Holloway, University of London, Egham TW20 0EX, UK; ^2^ Centre for Atmospheric Sciences, University of Manchester, Oxford Road, Manchester M13 9PL, UK; ^3^ National Centre for Atmospheric Sciences, Department of Chemistry, University of York, Heslington, York YO10 5DD, UK; ^4^ Laboratory for Atmospheric Physics, Institute for Physics Research, Universidad Mayor de San Andrés-UMSA, Campus Universitario, Cota-Cota Calle No 27, La Paz, Bolivia; ^5^ Facility for Airborne Atmospheric Measurement, Cranfield University, College Road, Cranfield MK43 0AL, UK; ^6^ 19 Jenkinson Road, Chisipite, Harare, Zimbabwe; ^7^ Geological Survey of Zambia, Ministry of Mines and Mineral Development, PO Box 50135, Ridgeway, Lusaka, Zambia; ^8^ Centre for Environment and Agricultural Informatics, Cranfield University, College Road, Cranfield MK43 0AL, UK; ^9^ Department of Earth Sciences, University of Oxford, South Parks Road, Oxford OX1 3AN, UK; ^10^ School of Geographical Sciences, University of Bristol, Bristol BS8 1SS, UK; ^11^ School of GeoSciences, University of Edinburgh, Edinburgh EH9 3FF, UK; ^12^ School of Marine and Atmospheric Sciences, Stony Brook University, Stony Brook, NY 11794, USA; ^13^ School of Chemistry, University of Bristol, Bristol BS8 1TS, UK; ^14^ Department of Chemistry, University of Cambridge, Lensfield Road, Cambridge CB2 1EW, UK; ^15^ Now at Jet Propulsion Laboratory, California Institute of Technology, Pasadena, CA 91109, USA; ^16^ British Antarctic Survey, Natural Environment Research Council, Cambridge CB3 0ET, UK; ^17^ Department Atmospheric and Oceanic Sciences, University of Maryland, College Park, MD 20742, USA; ^18^ Now at Faculty of Science and Engineering, University of Chester, Chester, UK; ^19^ Wolfson Atmospheric Chemistry Laboratories, Department of Chemistry, University of York, York YO10 5DD, UK; ^20^ National Centre for Earth Observation, University of Edinburgh, Edinburgh EH9 3FF, UK

**Keywords:** atmospheric methane, African wetlands, African biomass burning, African air pollution, methane isotopes, aircraft surveys

## Abstract

We report methane isotopologue data from aircraft and ground measurements in Africa and South America. Aircraft campaigns sampled strong methane fluxes over tropical papyrus wetlands in the Nile, Congo and Zambezi basins, herbaceous wetlands in Bolivian southern Amazonia, and over fires in African woodland, cropland and savannah grassland. Measured methane *δ*^13^C_CH_4__ isotopic signatures were in the range −55 to −49‰ for emissions from equatorial Nile wetlands and agricultural areas, but widely −60 ± 1‰ from Upper Congo and Zambezi wetlands. Very similar *δ*^13^C_CH_4__ signatures were measured over the Amazonian wetlands of NE Bolivia (around −59‰) and the overall *δ*^13^C_CH_4__ signature from outer tropical wetlands in the southern Upper Congo and Upper Amazon drainage plotted together was −59 ± 2‰. These results were more negative than expected. For African cattle, *δ*^13^C_CH_4__ values were around −60 to −50‰. Isotopic ratios in methane emitted by tropical fires depended on the C3 : C4 ratio of the biomass fuel. In smoke from tropical C3 dry forest fires in Senegal, *δ*^13^C_CH_4__ values were around −28‰. By contrast, African C4 tropical grass fire *δ*^13^C_CH_4__ values were −16 to −12‰. Methane from urban landfills in Zambia and Zimbabwe, which have frequent waste fires, had *δ*^13^C_CH_4__ around −37 to −36‰. These new isotopic values help improve isotopic constraints on global methane budget models because atmospheric *δ*^13^C_CH_4__ values predicted by global atmospheric models are highly sensitive to the *δ*^13^C_CH_4__ isotopic signatures applied to tropical wetland emissions. Field and aircraft campaigns also observed widespread regional smoke pollution over Africa, in both the wet and dry seasons, and large urban pollution plumes. The work highlights the need to understand tropical greenhouse gas emissions in order to meet the goals of the UNFCCC Paris Agreement, and to help reduce air pollution over wide regions of Africa.

This article is part of a discussion meeting issue 'Rising methane: is warming feeding warming? (part 2)'.

## 1. Introduction

The objectives were to measure methane in air over major tropical sources, especially African wetlands, regional agriculture and biomass burning, to determine at regional scale the characteristic isotopic signatures of these methane sources, and thereby to help constrain regional methane source fluxes and their roles in global methane budget.

There is strong evidence to suggest increasing tropical biological sources such as ruminants and wetlands are major drivers of methane's recent growth [1–4]. Growth in tropical methane emissions is consistent with a widening of regions experiencing tropical climate [5], land-use intensification and rapid population rise coupled with explosive urban growth.

The causes of the recent rapid growth in the atmospheric methane burden, and concurrent isotopic shift to values more depleted in 13C remain poorly understood [1,2,16]. Much of the current rise in the global methane burden is led from sources in the tropics [2–4].

Major tropical methane sources such as wetlands and cattle emit methane isotopically depleted in 13C compared to the bulk global source [2,3,34]. Methane emissions from tropical fires are also significant. But, though isotopic source signatures are key inputs needed if isotopic modelling is to help impose better constraints on global methane budgets, there have been very few studies of the isotopic signatures of methane sources emitting into tropical air masses, especially over central Africa.

Global methane budgets (e.g. [8]) are primarily ‘bottom-up’ aggregates of on-ground emissions estimates. They are unconstrained or only weakly constrained by isotopic balancing, a difficult task because isotopic data are very sparse from the tropics, especially the African tropics. The ‘top-down’ measurements reported here, made directly from the air or *in situ*, will allow better constraints to be placed on regional scale isotopic source signatures. In particular, methane emissions from tropical wetlands contribute 60–80% of global natural wetland CH_4_ emissions [9] but the carbon isotopic signatures (*δ*^13^C_CH_4__) of methane from African wetlands are very poorly known. Better understanding of African wetland and biomass burning *δ*^13^C_CH_4__ signatures will provide critical new data to constrain global isotopic inversions for methane.

Overall, Lunt *et al*. [10] estimated Africa's annual methane emissions between 2010 and 2016 to be around 76–80 Tg yr^−1^. This compares with total global emissions estimated at around 600 Tg (top down; [8]). Thus, to balance the global methane budget isotopically, understanding African and Amazonian emissions is critically important.

Hitherto most evidence for atmospheric emissions over tropical Africa has been from satellite remote sensing, or from model or desk studies. *In situ* direct measurement of the atmospheric boundary layer is rare in sub-Saharan Africa outside South Africa and Senegal [11]. Remote marine *in situ* observations, satellite remote sensing and measurement-linked modelling on a regional scale all imply very strong methane emissions from tropical regions in Africa and South America [3,10,12–16], but there have been very few direct measurements by well-instrumented aircraft and ground campaigns.

### (a) Isotopic signatures

Isotopic signatures are a critical input for using co-constrained isotopic mass balance modelling to understand the global methane budget [17,6]. For example, Schwietzke *et al*. [18] used isotopes to show that emissions from the fossil fuel industry (gas, oil and coal) were 20–60% greater than estimated in inventories.

Using isotopes to constrain global methane budgets and to understand the processes driving the current strong rise in the methane burden depends on having good information about *δ*^13^C_CH_4__ signatures of sources, especially tropical sources. But previously very few measurements have been made in the tropics [19], where much better measurement of *δ*^13^C_CH_4__ signatures is needed to assess wide-area wetland and fire inputs of methane into the ambient tropical air.

Thus the determination of regional *δ*^13^C_CH_4__ isotopic signatures of specific tropical methane sources is a key objective. Although a few *δ*^13^C_CH_4__ source signatures have been measured locally on the ground [20], regional-scale aircraft-based determinations of *δ*^13^C_CH_4__ signatures are lacking. In particular, low-altitude research aircraft flights such as those reported here permit integrated sampling of complex aggregations of emissions, difficult to assess by spot-sampling on the ground.

Tropical methane sources are diverse. They include emissions from wetlands, agriculture (especially from cattle, and crop waste burning) and large-area dry season fires (mostly human-lit), as well as emissions from the rapidly growing new urban population centres. An important factor that leads to locally distinctive *δ*^13^C_CH_4__ isotopic signatures is the metabolic make up of the local vegetation. Warm tropical grasslands, farms and wetlands are rich in C4 plants such as maize, sugar and papyrus and many pastoral grasses, with carbon contents that are comparatively rich in ^13^C. By contrast, trees, bushes and some grasses have C3 metabolisms, which discriminate highly against ^13^C.

Wetland vegetation in both tropical Africa and South America is typically dominated by C4 grasses, especially C4 papyrus in the equatorial zone, although C3 plants such as reeds are also widespread. Decay of rotting C4 organic debris emits methane with comparatively less negative *δ*^13^C_CH_4__ than methane from C3 vegetation. Although very little is known about emission mechanisms, it is likely that in wetlands rich in tall papyrus and reed stems, methane may be emitted not only through ebullition (which is then subject to isotopically fractionating methanotrophy in the water column) but also through plant and tree stem conduits. Thus on-surface chamber measurements may fail to capture accurately the *δ*^13^C_CH_4__ source signatures of emissions from areas with tall plants (like papyrus) and trees; instead, these signatures may be better captured by integrative aircraft sampling in low flights.

Agricultural methane sources in Africa are large and expected to grow further, driven by rapid growth in human populations and fertilizer use. Methane is produced both by farm ruminants and by crop waste burning. Sub-Saharan African ruminant populations (mainly cattle, but also goats and sheep) are very large [21,38,57]. Eructated *δ*^13^C_CH_4__ values in cattle breath depend on feed and pasture species, which are diverse—tropical cattle diets are typically rich in C4 pasture grasses and crop waste from C4 maize, millet, sorghum or sugar but also including C3 grasses, tree leaves and bushes. Biomass burning of crop waste is often of C4 crop plants like maize or sugar in moist regions, or millet waste in drier agriculture, although other crop waste includes C3 yams, sweet potato and palm waste, etc.

Dry season wildfires are widespread in Africa and South America. Incomplete combustion produces methane with *δ*^13^C_CH_4__ values that depend strongly and characteristically on the type of vegetation fuelling the fires (such as C4 grasses or C3 tree-leaf litter), and that typically has much more positive *δ*^13^C_CH_4__ than wetland emissions. In particular, grassland fires (dominantly C4 plants) tend to produce very ^13^C-rich methane, while methane in smoke from fires fuelled by C3 trees and leaf litter in facultatively deciduous woodland and forest tends to have rather more negative *δ*^13^C_CH_4__ values.

Africa's dense human populations, with fast growing large cites and major landfills, also emit methane. Fires, urban and rural village emissions cause significant local and regional air pollution in Africa [24], but there have been very few measurements of *δ*^13^C_CH_4__ in methane from these sources. Routine annual grass and crop waste fires, and widespread charcoal burning [25] lead to over 40 000 premature deaths annually from biomass burning aerosols [26] and there is poor air quality over wide areas of Africa [11,27,28]. Enhanced trace gas and particle abundances have been measured over major cities: Accra, Lomé, Abijan and Cotonou [29–31], but there have been few airborne campaigns over heavily populated and intensively farmed rural regions in equatorial Africa.

## 2. Methodology

As part of the UK Natural Environment Research Council's MOYA (The Global Methane Budget—Methane Observations and Yearly Assessment) and ZWAMPS (Quantifying methane emissions in remote tropical settings—Zambian Wetland Atmospheric Methane Production Study) projects, flight missions and associated on-the-ground field campaigns were carried out in Africa and South America. Flights in Africa used the NERC Facility for Airborne Atmospheric Measurement (FAAM) BAe-146 aircraft, flights in Bolivia used a Twin Otter aircraft operated by the British Antarctic Survey. The flights in Senegal, Uganda and Bolivia were supported by MOYA, and in Zambia by ZWAMPS. Analytical methods are documented in the electronic supplementary material, accessible online at rs.figshare.com.

### (a) Campaign locations: Senegal, Uganda, Zambia, Bolivia

Campaign locations are detailed in the electronic supplementary material, including maps and photographs.

The Senegal flights sampled winter fires in the Casamance region of southern Senegal in February/March (winter) 2017. The Casamance is a region of strongly seasonal rainfall, with a prolonged winter drought. The local vegetation [32] includes tropical woodlands (C3 rosewood), grazing land and seasonal cropland.

Uganda has a strong north-south variation of seasonality, climate, vegetation and agricultural types. The MOYA study had four distinct regional target areas:

(1) C4 Papyrus-dominated (*Cyperus papyrus*) (electronic supplementary material, figure SI 10) equatorial wetlands, including the Lake Wamala region which has both wetlands and widespread farming.
(2) Intensively cultivated agricultural central Uganda around Lake Kyoga. Crops include maize, finger millet, sorghum and sugar (C4), as well as cassava (C3–C4 intermediate), sweet potato (C3) and plantains (C3). There are also many cattle and extensive wetlands.
(3) C4 savannah grassland pastures in dry season northern Uganda.
(4) Regional background air over equatorial Lake Victoria (68 000 km^2^ area).

In Uganda, preparatory studies were carried out in 2014 in papyrus swamps on the shores of Lake Victoria between Kampala and Entebbe. The aircraft missions reported below were carried out on 24–29 January 2019. Flights were over several different terrains: (i) over equatorial wetlands, during the equatorial region's brief January dry season; (ii) over near-equatorial farming areas with both intensive crop farming and high cattle populations and (iii) over Northern Uganda in the winter dry season, sampling both woodland and savannah grass fires, by flying through large smoke plumes advected from active fires. Linked surface measurement campaigns on the ground took place both in preparatory work and also coincident with the aircraft flights. These campaigns accessed representative sources, with particular focus on Keeling plot determinations of papyrus wetland source signatures.

In Zambia, from 31 January to 4 February 2019 at the height of a very intense summer wet season [33], the main target was to investigate methane emissions from the large outer tropical wetlands. In particular, methane emissions from the Upper Congo basin [34] have had very little study and thus flights over the 11 000 km^2^ Bangweulu wetlands [35, 36] of Northern Zambia were the primary target. These very extensive wetlands, which are a major gathering centre for the Congo drainage, have dense C3 reed and C4 papyrus growth. In addition, flights were also carried out over the reed-rich Lukanga (central Zambia; [61]) and Kafue Flat (southern Zambia;[36]) wetlands in the Zambezi river drainage basin [34]. On the ground, sampling campaigns were carried out during the same week in the Lukanga wetlands and around Lusaka.

In addition, parallel on-ground sampling was carried out in Zimbabwe (fires, cattle, landfill) and for cattle in Kenya.

Bolivian sampling flights were over the Mamore River and Llanos de Moxos of North-East Bolivia [39,40], with simultaneous on-surface sample collection. A parallel paper in this collection [41] examines this region in more detail in the wider context of global tropical isotopic signatures from wetlands and rice fields.

### (b) Flight details

In Senegal, four survey flights were carried out: labelled C004–C007 (see electronic supplementary material, information). Transects through smoke plumes emanating from active fires were repeated at altitudes from 1000 ft (300 m) to 6000 ft (1800 m). See electronic supplementary material, figures SI 1 and 2 for flight paths, SI 3 for transect measurements, and [42] for further flight, instrumental and sampling details). Numerous fires were seen, some with large smoke plumes and visible fire fronts (electronic supplementary material, figure SI 4). Background conditions were determined by control flights over the Atlantic.

Flights along the coast of Senegal, Gambia, Guinea-Bissau and Guinea intersected multiple smoke plumes in the prevailing easterly wind, demonstrating that regional pollution was present, with very widespread smoke plumes in the boundary layer and lower free troposphere. Transport times of sampled smoke plumes ranged from a few minutes (in one plume overflown at low altitude over an active fire front (electronic supplementary material, figure SI 4)), to 9–12 h for plumes sampled over the ocean [43]. As there was no recent lightning from thunderstorms, fires were presumably human-lit, whether accidentally or deliberately.

In Uganda, flights using the FAAM aircraft were operated out of Entebbe Airport, Uganda (0° latitude), crossing the equator on take-off and landing. Flights took place during the long winter dry season in northern Uganda and during the brief early year relatively dry interval in the equatorial zone. Electronic supplementary material, figures SI 5–7 show flight paths and measurements, electronic supplementary material, figure SI 8 shows isotopic source attributions from plumes intersected in Kyoga transects, and electronic supplementary material, figure SI 9 shows an *in situ* Keeling plot sampled on the ground from a papyrus-dominated swamp at Kajjansi airstrip south of Kampala (electronic supplementary material, figure SI 10). Barker *et al.* [42] give further flight and sampling details.

In Zambia, FAAM flight surveys (electronic supplementary material, figure SI 11) took place in late January and early February 2019, at the height of the summer wet season [32]. Flight C136 over the Bangweulu wetlands (electronic supplementary material, figure SI 12) took place in a single fortunate dry day with very calm weather and vertical air advection during a very strong wet season with sustained heavy regional cloud cover over northern Zambia. Flights over the Lukanga and Kafue Flat wetlands were in a dry interval in a region of lower seasonal rainfall. Electronic supplementary material, figure SI 13a,b shows on-ground conditions in Lukanga swamp. Unfortunately, an aircraft problem cancelled a planned flight to determine emissions from the Lusaka metropolis.

In Bolivia, a few weeks after the Zambian campaign, flights were carried out in early March 2019, using the British Antarctic Survey's Twin Otter aircraft in the Amazonian Llanos de Moxos wetlands, in N.E. Bolivia, at similar latitude and climate setting to the Zambian campaigns. Further details are given by France *et al*. (2021-this volume; [41])

## 3. Results

### (a) Senegal fires—Casamance dry forest

**Figure 1.**
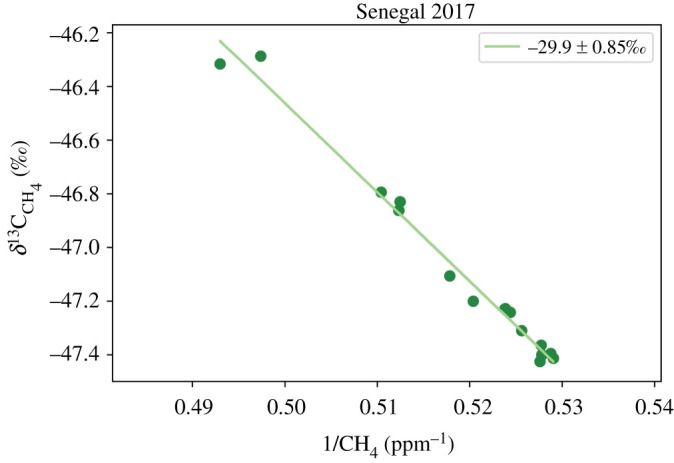
Keeling plot of (1/methane abundance) versus *δ*^13^C_CH_4__ for isotopic measurements of samples in a fire plume on FAAM flight C005 over the Senegal Casamance region. (Online version in colour.)

Keeling plot determination [44] of the methane increments in a smoke plume from Flight C005 gave source *δ*^13^C_CH_4__ about −29.9 ± 0.85‰ (figure 1), though varying with some plumes around −28‰, indicating the dominant fuel was C3 leaf litter, not C4 grasses, a finding consistent with the visual observation of burning forest litter. In smoke, enrichments of up to 0.5 ppm for CH_4_ and 300 ppb for CO were measured. Barker *et al*. [42] found these fires had mean emission factors units (in g per kg of dry fuel) of 1.8 ± 0.6 for CH_4_, 1630 ± 21.4 for CO_2_ and 67 ± 14 for CO, with a mean combustion efficiency of 0.94 ± 0.01, and obtained a *δ*^13^C_CH_4__ value of about −34‰ from all regional sources. Wu *et al*. [43] provide further details about the FAAM flights and sampling for the MOYA project, and report aerosol measurements and chemical transformations in biomass burning plumes sampled in the region.

### (b) Uganda—wetlands, savannah and farmlands

There was widespread visual and MODIS-satellite evidence of winter dry season grass fires during the flights over savannah northern Uganda. Significant local methane excesses sampled in air over northern Uganda's grasslands had incremental *δ*^13^C_CH_4__ around −16 to −12‰, indicating the methane sources were indeed C4 grass fires [45]. For these fires, Barker *et al*. [42] found mean emission factors (in g kg^−1^) of 3.1 ± 1.6 for CH_4_, 1610 ± 52.2 for CO_2_ and 78 ± 31 for CO, with a mean combustion efficiency of 0.93 ± 0.03. On one flight a mean N_2_O fire emission factor of 0.081 ± 0.020 g kg^−1^ was also measured.

Large methane increments were observed over the wetlands and agricultural districts of central Uganda (electronic supplementary material, figures SI 5,7). For fire plumes over Lake Kyoga, aircraft sampling found methane increments in individual plumes with *δ*^13^C_CH_4__ from −28 to −16‰ (electronic supplementary material, figure SI 8), suggesting the dominant fire fuel was C4 crop waste, such as maize, sorghum and millet, though in some fires perhaps admixed with cassava (C3–C4) or other C3 crop waste, or with emissions from the Kyoga wetlands.

**Figure 2.**
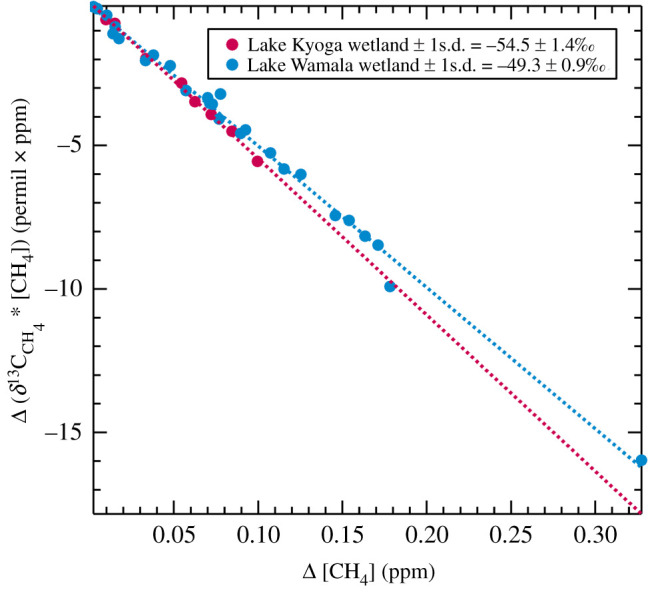
Miller–Tans plots of samples collected in regional air during flights over Lake Kyoga (*δ*^13^C_CH_4__ −54.5  ±  1.4‰) and Lake Wamala (*δ*^13^C_CH_4__ −49.3 ± 0.9‰), Uganda. (Online version in colour.)

A Miller–Tans plot (following the method of Miller & Tans [46]) of large methane increments (over background) measured in air over Lake Kyoga wetlands and neighbouring agricultural areas (figure 2 and electronic supplementary material, figure SI 7) had *δ*^13^C_CH_4__ of −54.5 ± 1.4‰. Methane in air over the Lake Wamala region of lake wetlands and surrounding farmlands in equatorial Uganda had *δ*^13^C_CH_4__ of −49.3 ± 0.9‰, indicating the methane came from complex mixed sources, likely including the wetlands, crop waste fires and ruminants [38] in this diverse and fertile region. These Miller–Tans plots likely represent regionally representative signals of methane inputs over these complex and varied landscapes.

A prior ground-based sampling campaign in Ugandan wetlands found *δ*^13^C_CH_4__ around −53.0 ± 0.4‰ for methane (electronic supplementary material, figure SI 9) from an equatorial papyrus swamp, though other samples from papyrus wetland near Kajjansi flanking Lake Victoria gave a poorly constrained value of −58.7 ± 4.1‰ [7]. *In situ* sampling was hand-held, and may have failed to access methane emitted from tall papyrus stem tops (3–5 m high) which had bypassed isotopically fractionating methanotrophic uptake in the water column.

Our wetland results compare with −61.2‰ and −62.2‰ values found by Tyler *et al*. [20] from Nyahururu marsh in Kenya. However, like our results over Lake Wamala, Tyler *et al*. [20] also found a range of values in other Kenyan wetlands, from −54‰ to −31‰, although with very high CO_2_ measurements in many samples, suggesting complex perturbation.

Our sampling from East African cattle, to be detailed elsewhere [47], found *δ*^13^C_CH_4__ around −57‰, a range comparable to −57 to −52‰ values we previously found in Zimbabwean cattle [7] and broadly similar to Australian results of −59.7 ± 0.7‰ from grazing cattle, and −62 : 9 ± 1 : 3‰ from feedlot cattle [48]. However, we note our results are significantly more ^13^C rich than the values around −65‰ found for sub-Saharan Africa by Chang *et al*. [49] (their fig. 4).

### (c) Zambia—Bangweulu, Kafue and Lukanga swamps

**Figure 3.**
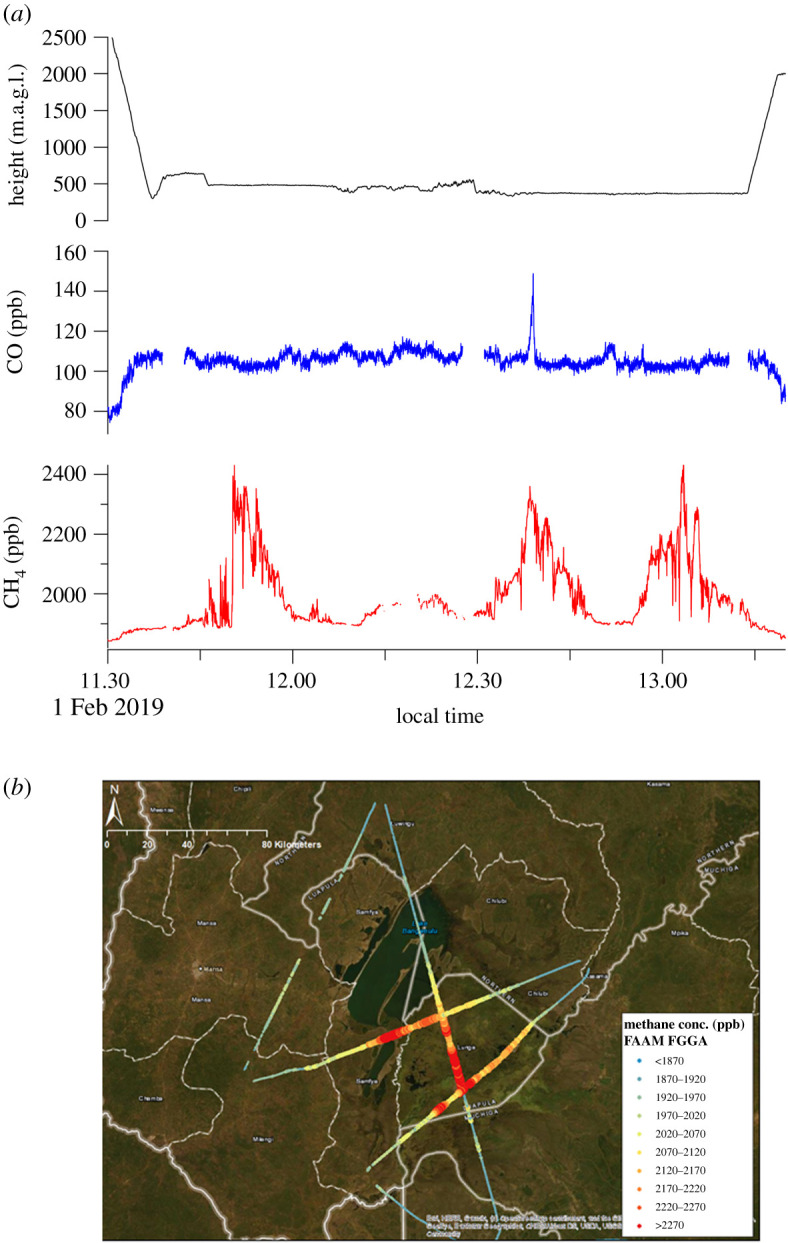
(*a*) ZWAMPS FAAM flight C136, height, CO and methane transects across Bangweulu wetlands. Height is metres above the ground surface. (*b*) ZWAMPS FAAM flight, showing measured methane abundance advected over the Bangweulu wetlands. Transects at various heights above ground level, coloured by *in situ* methane concentration as per legend. Note the highest values are over the wetlands SE of the lake, not over the large shallow lake. (Online version in colour.)

Strong methane emissions were observed over all the wetlands studied. The Bangweulu transects, flown in still weather conditions with vertical advection of air (see cloud in electronic supplementary material, figure SI 12), measured the highest values over wetlands, not the shallow lake (figure 3*a*,*b*). Isotopic results from 19 air samples collected on the FAAM aircraft over the Bangweulu wetlands found a very well constrained *δ*^13^C_CH_4__ source signature of −59.7 ± 1.3‰ for these Upper Congo wetlands. This may be the first such measurement from the Congo basin.

**Figure 4.**
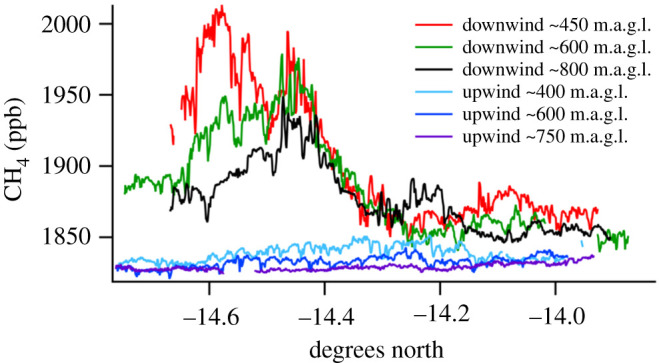
Lukanga swamp. Methane observations during flight transects at various heights above ground level. (Online version in colour.)

In the Kafue (Zambezi) basin, figure 4 shows upwind and downwind methane profiles at various altitudes around the Lukanga wetland, providing evidence for significant fluxes of methane from the swamp, perhaps up to 0.3 Tg annually [50]. Over Lukanga, 16 air samples collected on the aircraft gave *δ*^13^C_CH_4__ −62.1 ± 2.3‰.

Parallel on-ground sampling campaigns were also carried out along the margin of the Lukanga swamp, and cattle were widely observed in the wetlands. Unfortunately, the *in situ* isotopic determinations from Lukanga gave complex results, suggesting a diverse range of local sources advecting to the low flying aircraft. Similarly, diverse signatures have also been seen from ground-based work in the Okavango, Botswana [41] and may be related to local burning, variable methanotrophy or locally dominant plant species.

Flights over the third target, the Kafue Flat wetlands, also found substantial emissions with high local methane enhancements. Fire plumes were again observed, marked by elevated CO measurements and indicating a mixed source, although complex local meteorology during sampling of Kafue fluxes makes it difficult unambiguously to separate advected local sources from regional transport of emissions.

Over the Kafue Flats a Keeling plot of eleven samples collected on board the aircraft gave *δ*^13^C_CH_4__ −60.0 ± 1.2‰.

### (d) *δ*^13^C_CH_4__ results from the Mamore River basin, Llanos de Moxos, NE Bolivia

The Bolivian flights measured very large methane enhancements, from which a *δ*^13^C_CH_4__ source signature of −58.7‰ ± 1.9‰ was determined, with similar results from concurrent on-ground *in situ* sampling [41]

## 4. Interpretation of *δ*^13^C_CH_4__ results

### (a) Sahel fires

Prior to the flights over Senegal's southern Casamance region, the expectation had been that the fuel for most fires would be from tropical C4 grasses. That expectation was shown to be wrong from observation during the flights, when it was clear that the fires were primarily in forested areas. This observation was confirmed by the measured *δ*^13^C_CH_4__ −30‰ signature. This value, which is much more negative than likely from C4 grassfires, suggests the primary fuel was leaf litter and fallen or cut wood. The result also suggests that in addition to C4 grasses, C3 tree litter [51] may be a significant fuel for many of the very widespread winter fires across the West African Sahel. This observation is potentially important in the future use of isotopic data to model regional contributions to global methane growth.

### (b) Equatorial emissions

The complex isotopic results from aircraft sampling over central Uganda likely reflect the variety of sources over these rich densely populated agricultural regions, with wetlands, large cattle and other animal and human populations, and widespread crop waste and plastic waste fires. The −53‰ *δ*^13^C_CH_4__ values in air samples collected in on-foot fieldwork at water level from equatorial C4 papyrus swamps in Uganda (electronic supplementary material, figures SI 9 and 10) are consistent with the −49 to −55‰ values found in the Miller–Tans plots (figure 2*b*) of air samples collected in flights over the regions around Lake Kyoga and Lake Wamala. However, the relatively ^13^C-rich measurements over Lake Wamala likely reflect significant inputs from biomass burning.

### (c) Southern Hemisphere outer tropics

A Miller–Tans plot of *all* air samples collected over all three Zambian wetlands gave a *δ*^13^C_CH_4__ value of −59.8 ± 1.0‰ ([50] under review). The Zambian and Bolivian wetlands are very comparable. They are at approximately the same latitude in the outer tropics, and sampling was a few weeks apart during the later part of the rainy season in both places, when wetlands were filling. The Miller–Tans isotopic signature reported here from the outer tropical Upper Congo and Zambezi wetlands is very similar to −59‰ values of large methane fluxes measured in the comparable-latitude Bolivian Llanos de Moxos wetlands [41].

**Figure 5.**
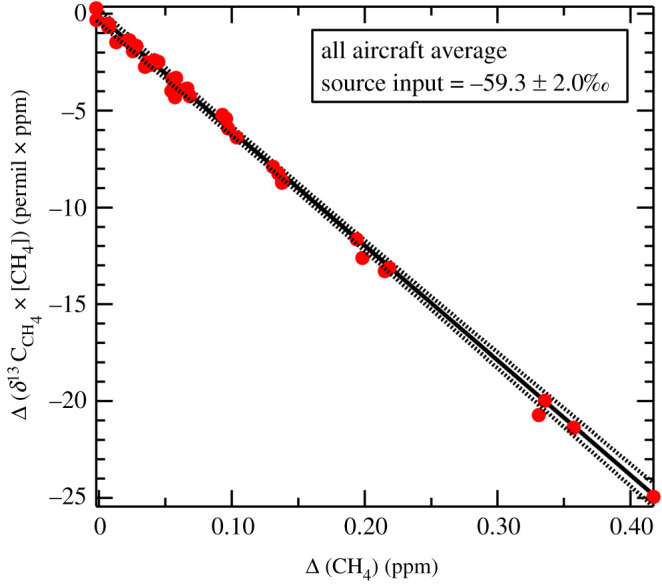
*δ*^13^C_CH_4__ signature of outer tropical wetlands of the Southern Hemisphere. Miller–Tans plot for data from Zambia and Bolivia. The inferred *δ*^13^C_CH_4__ value is −59.3 ± 2.0‰. Plot includes aircraft-collected samples from the Upper Congo (Bangweulu) and Zambezi basin (Lukanga, Kafue) wetlands in Zambia and from the Mamore River and Llanos de Moxos wetlands in Bolivian Amazonia. (Online version in colour.)

Given the similarity between the two regions, a Miller–Tans plot of all data from both areas is justified. Figure 5 shows that when the samples collected over Bolivian Amazonia were included with the Zambian data, the *δ*^13^C_CH_4__ value was −59.3 ± 2.0‰ (figure 5). As a first assumption, this value could be used in global isotopic modelling to represent the outer tropical Southern Hemisphere wetlands.

### (d) Mixed sources: how representative are the results?

Our data on East African and Zimbabwean cattle show that the *δ*^13^C_CH_4__ source signatures of African wetlands and ruminant emissions are probably indistinguishable. African wetland regions have significant animal populations, including cattle in the Lukanga swamp (electronic supplementary material, figure SI 13a), and also widespread antelopes (ruminants) and many hippoipotamoi (pseudo-ruminants). Thus the aircraft samples from African wetlands may also include significant eructated methane from ruminants and pseudo-ruminants.

The sampling areas flown over in Zambia and Bolivia were large and thus the overall −59‰ *δ*^13^C_CH_4__ value (figure 5) may be broadly representative of the seasonally moist outer tropical wetlands of both Africa and South America. This −59‰ outer tropical wetland signature is more depleted compared to our previous estimates of the bulk global atmospheric methane source at about −53‰ [13] and the −56.7‰ mean tropical signature used by Ganesan *et al*. [17]. However, these wetland results are comparable in range to our estimates of *δ*^13^C_CH_4__ around −55 to −60‰ emitted from grazing African and Australian cows.

**Figure 6.**
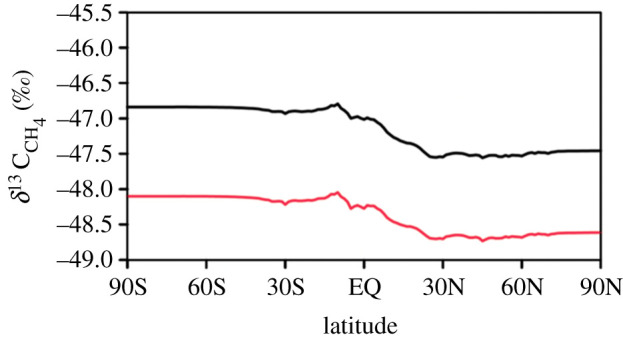
Global impact of changing the *δ*^13^C_CH_4__ source signature of methane emitted from tropical wetlands. Black line (upper line) is a model scenario optimized to NOAA observations with the tropical wetland source having a −55‰ *δ*^13^C_CH_4__ signature. Red line (lower line) shows the impact of changing the tropical wetland source *δ*^13^C_CH_4__ signature from −55‰ to −60‰ on the optimized model scenario, with nothing else varied. (Online version in colour.)

A possible explanation of the contrast between the −49 to −55‰ *δ*^13^C_CH_4__ values found in equatorial Uganda and the −59 ± 2‰ values measured in Zambia and Bolivia is that this may be seasonal, because the Ugandan campaign was carried out in equatorial Uganda's brief relatively dry season in January, and thus likely there was more ^13^C-rich methane from biomass burning than in wetter periods.

An alternative hypothesis is that the on-ground sampling in Uganda did not properly sample methane advected in papyrus swamps. Methanotrophy in water bodies is selective for ^12^CH_4_, and it is possible the relatively positive *δ*^13^C_CH_4__ values from the Ugandan on-ground samples, collected approximately 1 m above water level, record methane that is remaining after passing through a zone of methanotrophy during ebullition in the water, but that we failed to sample much less depleted methane channelled directly to the air from the high tops of the 3–5 m high papyrus stalks. By contrast, sampling from low flying aircraft collects bulk emissions and should be more representative of the bulk inputs.

However, a wider hypothesis for the greater ^13^C depletion measured by the flights in the outer tropics is that these more negative *δ*^13^C_CH_4__ values measured in flights over Zambia and Bolivia are consistent with a broad latitudinal C4 : C3 gradation in plant species, with C4 plants, especially papyrus, dominating in the equatorial wetlands, while in outer tropical Zambia, and in Bolivia, the proportion of C3 reeds and swamp grasses is higher [52].

## 5. Summary of isotopic signatures

Table 1 summarizes the results from this work and related studies published elsewhere.

## 6. Modelling

Wetlands are one of the largest global sources of atmospheric methane, estimated to contribute up to approximately 35% of global methane emissions (e.g. [8,53]), with the latitudinal gradient in atmospheric methane mole fractions observed in the NOAA network indicating the bulk of these emissions are situated in tropical rather than high latitude regions. Therefore, atmospheric *δ*^13^C_CH_4__ values predicted by global atmospheric models are sensitive to the *δ*^13^C_CH_4__ isotopic signature applied to tropical wetland emissions.

The evidence presented here shows a latitudinal range in *δ*^13^C_CH_4__ signatures of methane that actually enters the African troposphere, with equatorial emissions being less negative than −55‰, being in bulk derived from wetland vegetation, ruminant fodder and crop waste more rich in C4 species. By contrast, *δ*^13^C_CH_4__ signatures of African outer tropical emissions, from wetlands, pastures and farming somewhat richer in C3 plants, are closer to −60‰, which is similar to the results of the Bolivian measurements, at a latitude very similar to northern Zambia.

This finding has significant impact. Changing the tropical wetland *δ*^13^C_CH_4__ signature from −55‰ (the number currently adopted in many global model studies) to −60‰ in a global atmospheric model [54] resulted in a downward shift in the modelled global surface *δ*^13^C_CH_4__ atmospheric composition of approximately 1.2‰ at steady-state (figure 6). Similarly, adopting −60‰ as the bulk *δ*^13^C_CH_4__ isotopic signature of tropical wetland areas in the analysis of Ganesan *et al*. [17], which employed a different set of global methane fluxes from Warwick *et al*. [54], would shift the modelled global atmospheric *δ*^13^C_CH_4__ value by −0.5‰. Changes of this magnitude are large compared to the measured signals in atmospheric *δ*^13^C_CH_4__ values. Updating the tropical wetland *δ*^13^C_CH_4__ signature to −60‰ in model global budget studies would thus have an important impact on the methane source mixture that best fits the *δ*^13^C_CH_4__ observations.

While the magnitude of this impact may differ slightly between models depending on the source and sink assumptions, it represents a shift in *δ*^13^C_CH_4__, similar in magnitude to the shift resulting from uncertainties in the tropospheric chlorine sink [55]. Such a modelled shift is much larger than the observed shift in the global burden since 2007 [56]. Thus, the hypothesis that recent vegetation or land-use changes have made equatorial African wetlands emit methane that is isotopically more similar to outer tropical wetlands could in principle explain the post-2007 negative *δ*^13^C_CH_4__ shift in the global burden. This explanation is unlikely, as intuitively a warming climate would drive changes in the opposite direction, but is perhaps worth investigating.

**Table 1.** Summary of Isotopic Signatures. Senegal regional value from Barker *et al.* [42], Lake Victoria Swamp value from Brownlow *et al.* [7], Kenyan cattle from Cozens *et al*. [47], Zambia (all) from [50] (under review) and Bolivian wetlands from France *et al*. [41]. All other measurements from this work.

latitude	location	setting	type of vegetation	*δ*^13^C_CH_4__ (‰)
13° N	Senegal—Casamance	biomass burning	C3 woodlands	−29.9 ± 0.9
			leaf litter etc.	
13° N	Casamance	smoke plumes	C3 woodland	−28
13° N	Casamance	regional sources	woodland, arable	−34
3° N	N. Uganda	grassland	C4 fires	−16 to −12
1° N	Central Uganda	farmland fires	C4 and C4 fires	−28 to −16
1° N	Central Uganda Kyoga region	regional	C4 and C3 mixed wetlands and farming	−54.5 ± 1.4
0°	Central Uganda Wamala region	mixed wetlands and farming	C4 and C3	−49.3 ± 0.9
0°	Lake Victoria Wetlands	Kajjansi Swamp	C4 papyrus	−53.0 ± 0.4
0°	Lake Victoria Wetlands	swamp	C4 papyrus	−58.7 ± 4.1
1° S	Kenya	cattle	mixed fodder	around −57
11° S	Zambia—Bangweulu	wetlands	C4 and C3	−59.7 ± 0.7
			papyrus swamps	
14° S	Zambia—Lukanga	wetlands	C3 and C4	−62.1 ± 2.3
16° S	Zambia—Kafue	wetlands	C3 and C4	−60.0 ± 1.2
11–16° S	Zambia (all)	wetlands	C3 and C4	−59.8 ± 1.0
12–15° S	Bolivia	wetland flights	C3 and C4	−58.7 ± 1.9
	Zambia and Bolivia together	flights over wetlands	C3 and C4	−59.3 ± 2.0

More tropical measurement is needed, to determine the complex effects of seasonality, biomass burning and variations in cattle management and in the C3 : C4 metabolic make up of the surface vegetation [37]. Nevertheless, it is clear that increasing tropical wetland emissions may indeed be an important factor in the explanation of the current negative isotopic shift shown by the global burden [2,3].

## 7. Discussion

These aircraft and ground measurements have provided direct bulk evidence for the isotopic signature of methane emissions from moist tropical Africa and South America.

Tropical source regions have globally important methane emissions [1,2,3]. In particular, the regions sampled here have very large methane emissions. As part of this work, [50] (submitted) estimate the Bangweulu wetlands emissions to be around 1.2 Tg of methane annually and the smaller Lukanga swamp in excess of 0.3 Tg yr^−1^. The methane flux from the Bolivian Llanos de Moxos wetlands may be even greater than the Bangweulu emissions [41]. These very large fluxes are consistent with other estimates. For example, the Nile basin's Sudd wetlands [10,15], which are northern tropical Africa's equivalent of Bangweulu and have similar vegetation, may emit as much as 7 ± 3 Tg annually, although Lunt *et al*. [58] found a smaller flux: 3.5 Tg yr^−1^ in 2018–2019. To put these fluxes into context, they may be compared with total annual UK anthropogenic methane emissions around 2.1 Tg [59].

The magnitude of the Upper Congo Bangweulu fluxes imply the Congo basin, which includes many other similar wetland systems, many at lower and warmer altitudes than Bangweulu, contributes significantly to the isotopic balance of global methane emissions. Lunt *et al*. [10] estimate (their figure 4) that the Congo basin may emit 13 Tg yr^−1^ on average between 2010 and 2016. This number is consistent with a somewhat larger ‘guesstimate’ by comparison with the Amazon basin, which may emit very roughly 35–40 Tg of methane annually, depending on inter-annual variability, (e.g. see [60]), and if emissions are proportionate to area, the Congo basin, about half its size, would perhaps emit 17–20 Tg annually.

For biomass burning, the values measured and reported here illustrate the importance of identifying the fuel for the fires—whether from C3 plants, relatively richer in ^12^C, with *δ*^13^C_CH_4__ around −28‰, or from C4 grasses, relatively richer in ^13^C, with *δ*^13^C_CH_4__ around −16‰ to −12‰. However, what is clear from the flights is the complexity of the sources [61], with intense human activity in all regions where rainfall is adequate to support agriculture. Land surface modelling needs to address this: the sources are multiple and heavily dominated by the impact of human actions: cattle, crop fires, forest fires and C4 : C3 plant ratios all depend on humans.

The state of Africa's atmosphere and its greenhouse gas outputs and their likely responses to climate warming have had little attention: our work shows that this neglect needs to be rectified, particularly given the likely near-future growth in fossil fuel burning and vehicle emissions [62]. In many locations burning is uncontrolled, despite the widespread loss of agricultural nutrients into smoke, and air pollution is widespread in tropical Africa: all problems that demand attention. Although Africa's methane emissions are globally significant, national emissions inventories are as yet poorly constrained for the region. Desk studies are not enough; better measurement is needed. The novel isotopic source signatures for tropical wetlands and fires reported here represent important new co-constraints for use in global methane budget models. Further field measurements are urgently required to improve the representation of the tropics as a key global methane source region.

## Data accessibility

Data are archived in CEDA: the UK Centre for Environmental Data Analysis.
